# Astigmatism in school students of eastern China: prevalence, type, severity and associated risk factors

**DOI:** 10.1186/s12886-020-01425-w

**Published:** 2020-04-19

**Authors:** Jianyong Wang, Qianqian Ellie Cheng, Xiaojin Fu, Ronghua Zhang, Jia Meng, Fang Gu, Juanjuan Li, Gui-shuang Ying

**Affiliations:** 1 Department of Ophthalmology, The First Affiliated Hospital, College of Medicine, Zhejiang University, 79 Qingchun Road, Hangzhou, 310003 People’s Republic of China; 2 School of Arts and Sciences, University of Pennsylvania, Philadelphia, PA USA; 3 Department of Ophthalmology, Central Hospital of Yiwu City, Zhejiang, People’s Republic of China; 4 Center for Disease Prevention and Control, Hangzhou Zhejiang, People’s Republic of China; 5 Center for Preventive Ophthalmology and Biostatistics, Department of Ophthalmology, Perelman School of Medicine, University of Pennsylvania, Philadelphia, PA USA

## Abstract

**Background:**

China has been undergoing dramatic economic development, accompanied by increased education load on the young children. This study is to investigate the prevalence, type, severity, and associated risk factors of astigmatism in school students in eastern China.

**Method:**

In this cross-sectional school-based study, students underwent refraction using NIDEK non-cycloplegic autorefractor. Astigmatism was defined as cylinder 1.5 diopter (D) or greater, and high astigmatism was defined as cylinder 3.0 D or greaterMultivariate regression models were used to determine factors associated with astigmatism.

**Results:**

Among 4801 children (55% male) with mean age (±standard deviation) 12.3 (±3.8) years, 680 (14.2, 95% confidence interval (95% CI): 13.2–15.2%) had astigmatism (85% were with-the-rule) and 103 (2.2, 95% CI: 1.8–2.6%) had high astigmatism. The prevalence rate of astigmatism was 7–8% in grades 4 or below, 15–16% in grades 6–8, 20% in grade 9, and 20–25% in grade 10 or above. In multivariate analyses, higher grade and male gender were associated with higher prevalence of astigmatism (all *p* < 0.0001) and high astigmatism (*p* = 0.04 for grade, *p* = 0.001 for gender). When multivariate models were further adjusted by spherical equivalent, only gender remained statistically associated with astigmatism (odds ratio (OR) = 1.65, *p* < 0.0001) and high astigmatism (OR = 2.21, *p* = 0.0004), myopic and hyperopic refractive error were significantly associated with higher risk of astigmatism and high astigmatism (all *p* < 0.0001).

**Conclusion:**

Astigmatism is common in Chinese school-age children and increases with grade. Majority of astigmatism is with-the-rule. Male gender and myopic or hyperopic refractive error are significantly associated with higher prevalence and severity of astigmatism.

## Background

Astigmatism is a common vision disorder in children. Previous studies suggested that uncorrected astigmatism was associated with increased risk of myopia and amblyopia [1–4]. Early detection and treatment of astigmatism in children is important because of its potential influence on normal vision development. The exact cause of astigmatism in children is unknown. Studies in young children of United States identified risk factors of astigmatism including younger age, Hispanic ethnicity, African American race, presence of significant refractive error (myopia or hyperopia), and maternal smoking during pregnancy [4, 5]. In recent years, China has been undergoing dramatic economic development, accompanied by increased education load on the young children that results in high myopia prevalence rate, particularly in well-developed urban area. These changes may have substantial impact on the prevalence of astigmatism. The purpose of this study is to evaluate the prevalence, type and severity of astigmatism and the associated risk factors in Chinese school-age children in a well-developed Yiwu city of eastern China.

## Methods

This is a cross-sectional school-based study of refractive error conducted in May 2019 in YiWu, a county-level city of about 1.2 million people in eastern China of central Zhejiang province [6]. For this study, a simple random sample of 18 schools including kindergarten, elementary school (grade 1 to 6), middle school (grade 7 to 9) and high school (grade 10 to 12) was selected. Among the selected schools, simple random samples of classes from each grade were selected and all students from the selected classes were invited to participate the study so that at least 80 students from each grade of the selected schools were enrolled into study.

The information on student name, date of birth, gender and grade were obtained from the school roster. All participants underwent eye examination following the standard study protocol by the trained eye-care professionals (optometrists or ophthalmologists) for common ocular diseases, tests for the distance visual acuity using retro-illuminated logMAR chart with tumbling-E optotypes followed by measuring for refractive error using table-mounted NIDEK noncycloplegic autorefractor (Model: AR-1 s, Japan). Three readings of refractive error were taken from each eye and the average of three readings for each eye was entered for analysis. If the difference between any of two readings from an eye was greater than 0.5 diopters, refractive error for that eye was re-taken. Students who were found having ocular diseases (pediatric cataract, glaucoma, optic neuropathy) or ocular injuries other than the significant refractive error were not eligible for the study.

For quality control, autorefractors were calibrated every day before measuring refractive error. Approximately 5% of students were randomly chosen to repeat the test of refraction. The data were double entered into excel sheets and the differences were resolved by checking with the original paper record.

The study was designed to enroll 4800 students. This sample size provides very precise estimate of prevalence rate of astigmatism with half width of its 95% confidence interval 1% assuming prevalence rate of astigmatism is 10%.

The study was approved by the Institutional Review Board, and written informed consent was obtained from at least one parent or legal guardian.

### Statistical analysis

Spherical equivalent (SE) for each eye was calculated as sphere plus half of the cylinder. Cylindrical refractive error was expressed as positive cylinder form. To facilitate the direct comparison with other studies [4, 5], we defined presence of astigmatism as cylinder power 1.5 diopters (D) or greater in either eye and defined high astigmatism as cylinder power 3.0 D or greater in either eye. Children with astigmatism were further classified into three types of astigmatism including with-the-rule (plus cylinder axis 90 ± 15 degrees), against-the-rule (plus cylinder axis 180 ± 15 degrees) and oblique (plus cylinder axis 15 to 75 degrees or 105 to 165 degrees). When both eyes had astigmatism, the eye with higher cylinder power was used for classifying the severity and type of the astigmatism.

Risk factors for magnitude of cylinder power (among all children and among those with astigmatism) were assessed using analysis of variance, and comparisons of prevalence rates of astigmatism and high astigmatism across levels of risk factors were performed using the chi-square test. To evaluate the independent association of the each risk factors (grade, gender, school location) with cylinder power and prevalence of astigmatism, multivariate linear regression models were performed for cylinder power, and multivariate logistic regression models were performed for prevalence of astigmatism. The odds ratio (OR) and its 95% confidence interval (95% CI) for each risk factors were calculated from the multivariate logistic regression models. In the multivariate regression models, the grade instead of age was used, because age and grade were highly correlated (Pearson correlation coefficient of 0.99) and grade was slightly more associated with astigmatism than age. We performed two multivariate models, the multivariate model 1 included grade, gender and school location, while the multivariate model 2 additionally included spherical equivalent (grouped into levels of myopia, emmetropia and hyperopia), to assess whether the potential associations of grade, gender and school location with astigmatism were due to the spherical equivalent.

All statistical analyses were performed in SAS v9.4 and two-sided *p* < 0.05 was considered to be statistically significant.

## Results

### Prevalence of astigmatism

A total of 4801 school-aged students from 16 schools of YiWu city participated in the study. The student grade ranged from kindergarten to grade 12 with the number of participating students ranging from 331 to 403 in each grade (Table 1). The mean age (± standard deviation) was 12.3 (±3.8) years ranging from 5 to 20 years, 2647 (55.1%) were male, and 2691 (56.1%) were from urban schools (Table 1). The median cylinder power was 0.75 diopter (D) (inter-quartile: 0.5 to 1.12) ranging from 0 to 15.0 D. Among all 4801 students, 680 (14.2, 95% CI: 13.2–15.2%) had astigmatism of 1.5 D or greater, and 103 (2.2, 95% CI: 1.8–2.6%) had high astigmatism of 3.0 D or greater. Among 680 students with astigmatism of 1.5 D or greater, 575 (84.6%) were with-the-rule, 11 (1.6%) were against-the-rule, and 94 (13.8%) were oblique. Among 103 students with high astigmatism, 93 (90.3%) were with-the-rule, 2 (1.9%) were against-the-rule, and 8 (7.8%) were oblique (Table 1).

**Table 1:** Characteristics of study participants (*N* = 4801)

Participant characteristics	
Age (years)	n (%)
5	1 (0.02%)
6	292 (6.1%)
7	345 (7.2%)
8	359 (7.5%)
9	369 (7.7%)
10	360 (7.5%)
11	385 (8.0%)
12	353 (7.3%)
13	374 (7.8%)
14	365 (7.6%)
15	371 (7.7%)
16	361 (7.5%)
17	370 (7.7%)
18	370 (7.7%)
19	121 (2.5%)
20	5 (0.1%)
Mean (SD)	12.3 (3.8)
Gender	
Male	2647 (55.1%)
Female	2154 (44.9%)
Grade	
Kindergarten	381 (7.9%)
Grade 1	331 (6.9%)
Grade 2	366 (7.6%)
Grade 3	371 (7.7%)
Grade 4	367 (7.6%)
Grade 5	376 (7.8%)
Grade 6	368 (7.7%)
Grade 7	376 (7.8%)
Grade 8	360 (7.5%)
Grade 9	346 (7.2%)
Grade 10	383 (8.0%)
Grade 11	373 (7.8%)
Grade 12	403 (8.4%)
School location	
Urban	2691 (56.1%)
Rural	2110 (43.9%)
Spherical equivalent of more astigmatic eye (Diopters)	
≤ −6	351 (7.3%)
> −6, ≤ −5	267 (5.6%)
> −5, ≤ −4	414 (8.6%)
> −4, ≤ −3	510 (10.6%)
> −3, ≤ −2	584 (12.2%)
> −2, ≤ −1	768 (16.0%)
> −1, ≤ −0.5	557 (11.6%)
> −0.5, < 0.5	1076 (22.4%)
≥ 0.5, < 1.0	171 (3.6%)
≥ 1.0	103 (2.2%)
Cylinder of more astigmatic eye (diopters)	
0	204 (4.3%)
> 0, < 0.5	952 (19.8%)
≥ 0.5, < 1.0	2063 (43.0%)
≥ 1.0, < 1.5	902 (18.8%)
≥ 1.5, < 2.0	349 (7.3%)
≥ 2.0, < 2.5	162 (3.4%)
≥ 2.5, < 3.0	66 (1.4%)
≥ 3.0, < 4.0	73 (1.5%)
≥ 4.0	30 (0.6%)
Mean (SD)	0.87 (0.75)
Median (1st quartile, 3rd quartile)	0.75 (0.50, 1.12)
Minimum, Maximum	0, 15
**Astigmatism ≥ 1.5 diopters**	680 (14.2%)
With-the-rule	575 (84.6%)
Against-the-rule	11 (1.62%)
Oblique	94 (13.8%)
**Astigmatism ≥ 3.0 diopters**	103 (2.15%)
With-the-rule	93 (90.3%)
Against-the-rule	2 (1.94%)
Oblique	8 (7.77%)

*SD* Standard deviation

**Fig. 1:**
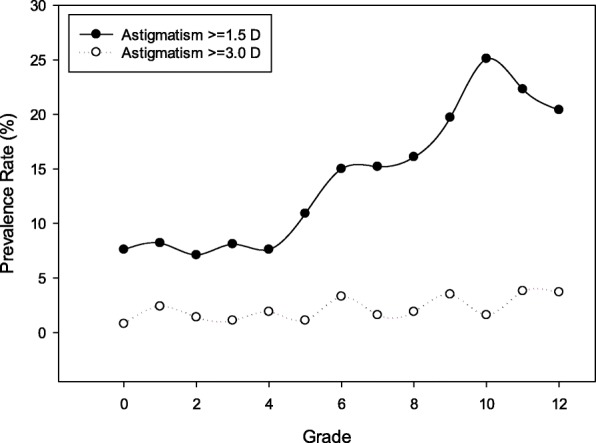
Prevalence rate of astigmatism (≥ 1.5 diopters) and high astigmatism (≥ 3.0 diopters) by grade from kindergarten (grade 0) to grade 12

The prevalence rate of astigmatism was 7–8% in kindergarten and grade 1 to 4, increased to 15–16% in grade 6–8, and 20% to grade 9, and 20–25% in grade 10 or above (Table 2, Fig. 1). The prevalence rate of high astigmatism was 0.8% in kindergarten, 1.8% in elementary school, 2.3% in middle school and 3.0% for high school (Table 2, Fig. 1).

**Table 2:** Magnitude of cylinder power and prevalence rate of astigmatism by characteristics of students (*N* = 4804)

Characteristics	N	Cylinder power in diopters: Mean (SD)	Astigmatism ≥1.5 D (%)	Astigmatism ≥3.0 D (%)	Degree of astigmatism (D) among students with astigmatism (*N* = 680) Mean (SD)
Age (years)		*P* < 0.0001	*P* < 0.0001	*P* = 0.02	*P* = 0.81
≤ 6	293	0.69 (0.61)	25 (8.5%)	3 (1.0%)	2.17 (0.97)
7	345	0.64 (0.57)	25 (7.3%)	6 (1.7%)	2.19 (0.79)
8	359	0.73 (0.76)	27 (7.5%)	5 (1.4%)	2.24 (0.94)
9	369	0.73 (0.58)	30 (8.1%)	5 (1.4%)	2.17 (0.72)
10	360	0.71 (0.59)	24 (6.8%)	6 (1.7%)	2.26 (1.07)
11	385	0.78 (0.85)	40 (10.4%)	5 (1.3%)	2.08 (0.65)
12	353	0.96 (0.93)	54 (15.3%)	12 (3.4%)	2.37 (0.94)
13	374	0.89 (0.62)	56 (15.0%)	7 (1.9%)	2.04 (0.62)
14	365	0.90 (0.63)	56 (15.3%)	4 (1.1%)	2.05 (0.64)
15	371	1.02 (0.78)	67 (18.1%)	14 (3.8%)	2.36 (0.82)
16	361	1.09 (1.03)	93 (25.8%)	5 (1.4%)	2.17 (1.48)
17	370	1.07 (0.86)	76 (20.5%)	11 (3.0%)	2.27 (0.88)
18	370	1.07 (0.87)	85 (23.0%)	15 (4.1%)	2.26 (1.04)
≥ 19	126	1.06 (0.79)	22 (17.5%)	5 (4.0%)	2.36 (0.95)
Gender		*P* < 0.0001	*P* < 0.0001	*P* = 0.001	*P* = 0.04
Male	2647	0.92 (0.89)	427 (16.1%)	73 (2.8%)	2.27 (1.06)
Female	2154	0.82 (0.80)	253 (11.8%)	30 (1.4%)	2.13 (0.78)
Grade		*P* < 0.0001	P < 0.0001	P = 0.02	*P* = 0.88
Kindergarten	381	0.67 (0.58)	29 (7.6%)	3 (0.8%)	2.16 (0.91)
Grade 1	331	0.66 (0.65)	27 (8.2%)	8 (2.4%)	2.33 (0.95)
Grade 2	366	0.74 (0.71)	26 (7.1%)	5 (1.4%)	2.14 (0.79)
Grade 3	371	0.73 (0.57)	30 (8.1%)	4 (1.1%)	2.16 (0.69)
Grade 4	367	0.72 (0.62)	28 (7.6%)	7 (1.9%)	2.27 (1.11)
Grade 5	376	0.82 (0.84)	41 (10.9%)	4 (1.1%)	2.03 (0.56)
Grade 6	368	0.93 (0.92)	56 (15.0%)	12 (3.3%)	2.35 (0.96)
Grade 7	376	0.91 90.61)	57 (15.2%)	6 (1.6%)	2.05 (0.55)
Grade 8	360	0.94 (0.71)	58 (16.1%)	7 (1.9%)	2.17 (0.85)
Grade 9	346	1.01 (0.75)	68 (19.7%)	12 (3.5%)	2.25 (0.73)
Grade 10	383	1.10 (1.02)	96 (25.1%)	6 (1.6%)	2.22 (1.48)
Grade 11	373	1.09 (0.89)	83 (22.3%)	14 (3.8%)	2.29 (0.86)
Grade 12	403	1.06 (0.83)	82 (20.4%)	15 (3.7%)	2.26 (1.06)
Grade levels		*P* < 0.0001	*P* < 0.0001	*P* = 0.03	*P* = 0.66
Kindergarten	381	0.66 (0.58)	29 (7.6%)	3 (0.8%)	2.16 (0.91)
Elementary school (grade 1–6)	2179	0.75 (0.63)	207 (9.5%)	40 (1.8%)	2.22 (0.86)
Middle school (grade 7–9)	1082	0.95 (0.69)	183 (16.9%)	25 (2.3%)	2.16 (0.72)
High school (grade 10–12)	1159	1.08 (0.90)	261 (22.5%)	35 (3.0%)	2.25 (1.18)
School location		*P* < 0.0001	*P* = 0.0001	*P* = 0.29	*P* = 0.64
Urban	2691	0.91 (0.76)	427 (15.9%)	63 (2.3%)	2.20 (1.00)
Rural	2110	0.82 (0.69)	253 (12.0%)	40 (1.9%)	2.24 (0.91)
Spherical equivalent of more astigmatic eye (diopters)		*P* < 0.0001	*P* < 0.0001	*P* < 0.0001	*P* = 0.008
≤ −6	351	1.67 (1.26)	174 (49.6%)	33 (9.4%)	2.46 (1.36)
> −6, ≤ −5	267	1.23 (0.81)	75 (28.1%)	12 (4.5%)	2.21 (0.87)
> −5, ≤ −4	414	1.07 (0.66)	90 (21.7%)	10 (2.4%)	2.05 (0.61)
> −4, ≤ −3	510	0.96 (0.68)	84 (16.5%)	10 (2.0%)	2.12 (0.84)
> −3, ≤ −2	584	0.78 (0.61)	54 (9.3%)	7 (1.2%)	2.18 (0.93)
> −2, ≤ −1	768	0.75 (0.61)	67 (8.7%)	14 (1.8%)	2.26 (0.79)
> −1, ≤ −0.5	557	0.68 (0.47)	35 (6.3%)	3 (0.5%)	1.96 (0.52)
> −0.5, < 0.5	1076	0.62 (0.47)	48 (4.5%)	6 (0.7%)	2.05 (0.78)
≥ 0.5, < 1.0	171	0.69 (0.56)	20 (11.7%)	0 (0.0%)	1.87 (0.42)
≥ 1.0	103	1.23 (0.92)	33 (32.0%)	8 (7.8%)	2.32 (0.72)

### Factors associated with cylinder power and prevalence of astigmatism

In univariate analysis (Table 2), older age, male gender, higher grade and urban school were significantly associated with larger mean cylinder power and higher prevalence rate of astigmatism (all *p* < 0.001), while older age (*p* = 0.02), male gender (*p* = 0.001), higher grade (p = 0.02) were significantly associated with higher prevalence rate of high astigmatism, but school location was not associated with high astigmatism (*p* = 0.29).

**Fig. 2:**
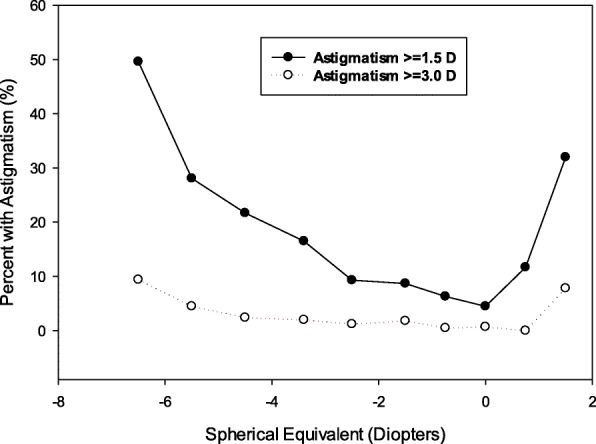
Prevalence rate of astigmatism (≥ 1.5 diopters) and high astigmatism (≥ 3.0 diopters) by magnitude of spherical equivalent

Magnitude of spherical equivalent (categorized into levels of myopia, emmetropia and hyperopia) was significantly associated with cylinder power, prevalence rates of astigmatism and high astigmatism in a non-linear manner (all *P* < 0.0001), with higher rates of astigmatism and high astigmatism in myopic and hyperopic children, and lower rates in emmetropic children (Table 2, Fig. 2).

In multivariate analysis for cylinder power, higher grade (*p* < 0.0001), male gender (*p* < 0.0001) were independently associated with higher cylinder power (Table 3). However, when the multivariate model was further adjusted by spherical equivalent, only male gender (*p* < 0.0001) and spherical equivalent (*p* < 0.0001) were significantly associated with higher cylinder power; the grade (*p* = 0.54) and school location (*p* = 0.18) were not significantly associated with cylinder power.

**Table 3:** Multivariate analyses for factors associated with cylinder power among all students (*N* = 4801)

		Multivariate Model 1: without including spherical equivalent*		**Multivariate Model 2: including spherical equivalent**^**§**^	
Characteristics	N	Mean Cylinder in diopter (SE)	*P*-value	Mean Cylinder in diopter (SE)	*P*-value
Grade			< 0.0001		0.54
Kindergarten	381	0.66 (0.04)		0.91 (0.04)	
Grade 1	331	0.65 (0.04)		0.92 (0.04)	
Grade 2	366	0.70 (0.04)		0.94 (0.04)	
Grade 3	371	0.72 (0.04)		0.94 (0.04)	
Grade 4	367	0.71 (0.04)		0.93 (0.04)	
Grade 5	376	0.79 (0.04)		0.94 (0.04)	
Grade 6	368	0.90 (0.04)		1.01 (0.04)	
Grade 7	376	0.90 (0.04)		0.98 (0.04)	
Grade 8	360	0.93 (0.04)		0.97 (0.04)	
Grade 9	346	1.01 90.04)		1.01 (0.04)	
Grade 10	383	1.09 (0.04)		1.02 (0.04)	
Grade 11	373	1.06 (0.04)		0.98 (0.04)	
Grade 12	403	1.04 (0.04)		0.93 (0.04)	
Gender			< 0.0001		< 0.0001
Male	2647	0.91 (0.01)		1.01 (0.02)	
Female	2154	0.81 (0.02)		0.91 (0.02)	
School location			0.10		0.18
Urban	2691	0.88 (0.02)		0.97 (0.02)	
Rural	2110	0.84 (0.01)		0.95 (0.02)	
Spherical equivalent of more astigmatic eye (Diopters)					< 0.0001
≤ −6	351			1.64 (0.04)	
> −6, ≤ −5	267			1.21 (0.04)	
> −5, ≤ −4	414			1.04 (0.04)	
> −4, ≤ −3	510			0.94 (0.03)	
> −3, ≤ −2	584			0.77 (0.03)	
> −2, ≤ −1	768			0.74 (0.03)	
> −1, ≤ −0.5	557			0.68 (0.03)	
> −0.5, < 0.5	1076			0.63 (0.02)	
≥ 0.5, < 1.0	171			0.71 (0.06)	
≥ 1.0	103			1.23 (0.07)	

*Multivariate model 1 included grade, gender and school location

^**§**^Multivariate model 2 included grade, gender, school location and spherical equivalent

In multivariate analysis (Table 4) for prevalence of astigmatism, higher grade and male gender were independently associated with higher prevalence rate of astigmatism (*p* < 0.0001). When the multivariate analysis were further adjusted by spherical equivalent, male gender remained significantly associated with higher prevalence of astigmatism (OR = 1.65, *p* < 0.0001), but grade became not significantly associated with astigmatism (*p* = 0.65). Myopic spherical equivalent (OR = 20.3 for myopia − 6 D or greater) or hyperopic spherical equivalent (OR = 10.3 for hyperopic 1.0 D or greater) were significantly associated higher prevalence rate of astigmatism when compared with emmetropic spherical equivalent (− 0.5 to 0.5 D).

**Table 4:** Multivariate analyses for factors associated with astigmatism ≥1.5 diopters (*N* = 4801)

	**Multivariate Model 1: without including spherical equivalent** *		**Multivariate Model 2: including spherical equivalent**^**§**^	
Characteristics	Odds ratio (95% CI)	*P*-value	Odds ratio (95% CI)	*P*-value
Grade		< 0.0001		0.65
Kindergarten	Reference		Reference	
Grade 1	1.08 (0.63, 1.87)		1.13 (0.64, 2.00)	
Grade 2	0.94 (0.54, 1.64)		0.91 (0.51, 1.62)	
Grade 3	1.09 (0.64, 1.85)		0.96 (0.55, 1.68)	
Grade 4	1.03 (0.60, 1.77)		0.94 (0.53, 1.68)	
Grade 5	1.55 (0.94, 2.56)		1.05 (0.60, 1.81)	
Grade 6	2.16 (1.35, 3.55)		1.26 (0.74, 2.15)	
Grade 7	2.22 (1.38, 3.55)		1.16 (0.67, 1.95)	
Grade 8	2.37 (1.48, 3.81)		1.07 (0.61, 1.79)	
Grade 9	3.06 (1.92, 4.85)		1.26 (0.73, 2.10)	
Grade 10	4.06 (2.60, 6.35)		1.41 (0.82, 2.31)	
Grade 11	3.55 (2.26, 5.59)		1.15 (0.69, 1.96)	
Grade 12	2.98 (1.89, 4.69)		0.90 (0.74, 1.07)	
Gender		< 0.0001		< 0.0001
Male	1.50 (1.27, 1.78)		1.65 (1.39,1.98)	
Female	Reference		Reference	
School location		0.11		0.18
Urban	1.15 (0.97, 1.38)		1.14 (0.94, 1.36)	
Rural	Reference		Reference	
Spherical equivalent of more astigmatic eye (Diopters)				< 0.0001
≤ −6			20.3 (13.5, 30.6)	
> −6, ≤ −5			8.05 (5.18, 12.7)	
> −5, ≤ −4			5.48 (3.68, 8.47)	
> −4, ≤ −3			4.00 (2.69, 6.10)	
> −3, ≤ −2			2.12 (1.45, 3.42)	
> −2, ≤ −1			2.05 (1.39, 3.10)	
> −1, ≤ −0.5			1.42 (0.90, 2.24)	
> −0.5, < 0.5			Reference	
≥ 0.5, < 1.0			2.94 (1.69, 5.12)	
≥ 1.0			10.3 (6.16, 17.1)	

*Multivariate model 1 included grade, gender and school location

^**§**^Multivariate model 2 included grade, gender, school location and spherical equivalent

In multivariate analysis for high astigmatism (Table 5), higher grade (*p* = 0.04), male gender (*p* = 0.001) were independently associated with higher prevalence rate of high astigmatism, while school location (*p* = 0.63) was not associated with high astigmatism. When spherical equivalent was included in the multivariate model, only male gender (OR = 2.21, *p* = 0.0004) and myopic (OR = 29.6 for spherical equivalent − 6 or worse) or hyperopic spherical equivalent (OR = 5.4 for hyperopia 0.5 or greater) were significantly associated with high astigmatism.

**Table 5:** Multivariate analyses for the factors associated with astigmatism ≥3.0 diopters (*N* = 4801)

	Multivariate Model 1: without including spherical equivalent *		**Multivariate Model 2: including spherical equivalent**^**§**^	
Characteristics	Odds ratio (95% CI)	*P*-value	Odds ratio (95% CI)	*P*-value
Grade		0.04		0.17
Kindergarten	Reference		Reference	
Grade 1	3.15 (0.83, 12.0)		3.11 (0.80, 11.9)	
Grade 2	1.79 (0.42, 7.53)		1.61 (0.37, 6.97)	
Grade 3	1.41 (0.31, 6.33)		1.13 (0.24, 5.29)	
Grade 4	2.55 (0.65, 9.95)		2.04 (0.49, 8.47)	
Grade 5	1.45 (0.32, 6.63)		0.78 (0.16, 3.78)	
Grade 6	4.33 (1.21, 15.5)		2.03 (0.52, 7.80)	
Grade 7	2.11 (0.52, 8.47)		0.86 (0.19, 3.83)	
Grade 8	2.56 (0.66, 9.97)		0.86 (0.20, 3.72)	
Grade 9	4.69 (1.31, 16.8)		1.47 (0.36, 5.91)	
Grade 10	2.05 (0.51, 8.32)		0.48 (0.11, 2.19)	
Grade 11	5.23 (1.48, 18.4)		1.22 (0.30, 4.87)	
Grade 12	4.71 (1.34, 3.12)		1.05 (0.26, 4.18)	
Gender		0.001		0.0004
Male	2.03 (1.32, 3.12)		2.21 (1.43, 3.42)	
Female	Reference		Reference	
School location		0.63		0.69
Urban	1.11 (0.73, 1.68)		1.09 (0.71, 1.66)	
Rural	Reference		Reference	
Spherical equivalent of more astigmatic eye (Diopters)				< 0.0001
≤ −6			29.6 (10.7, 82.2)	
> −6, ≤ −5			13.3 (4.39, 40.2)	
> −5, ≤ −4			6.37 (2.59, 24.7)	
> −4, ≤ −3			5.19 (1.72, 15.7)	
> −3, ≤ −2			2.83 (0.89, 9.01)	
> −2, ≤ −1			4.14 (1.52, 11.2)	
> −1, ≤ −0.5			1.03 (0.25, 4.17)	
> −0.5, < 0.5			Reference	
≥ 0.5			5.44 (1.86, 15.9)	
	AUC = 0.66 (0.61, 0.72)		AUC = 0.79 (0.74, 0.83)	

*Multivariate model 1 included grade, gender and school location

^§^Multivariate model 2 included grade, gender, school location and spherical equivalent

### Factors associated with type and severity of astigmatism

In univariate analysis, age, gender, grade, school location and spherical equivalent were all not significantly associated with type of astigmatism (all *p* ≥ 0.09, online supplement Table 6).

**Table 6:** (Online supplement): Univariate analysis for factors associated with type of astigmatism among those with astigmatism (*N* = 680)

		**Type of astigmatism**			
Demographics	# of students with astigmatism	With-the-rule n (%)	Against-the-rule n (%)	Oblique n (%)	*P*-value
Age (years)					0.37
≤ 6	25	20 (80%)	2 (8%)	3 (12%)	
7	25	22 (88%)	1 (4%)	2 (8%)	
8	27	23 (85%)	0 (0%)	4 (15%)	
9	30	27 (90%)	1 (3%)	2 (7%)	
10	24	23 (96%)	0 (0%)	1 (4%)	
11	40	32 (80%)	0 (0%)	8 (20%)	
12	54	47 (87%)	0 (0%)	7 (13%)	
13	56	48 (86%)	0 (0%)	8 (14%)	
14	56	47 (84%)	0 (0%)	9 (16%)	
15	67	57 (85%)	0 (0%)	10 (15%)	
16	93	81 (87%)	1 (1%)	11 (12%)	
17	76	65 (86%)	3 (3%)	9 (12%)	
18	85	66 (78%)	4 (5%)	15 (18%)	
≥ 19	22	17 (77%)	0 (0%)	5 (23%)	
Grade					0.39
Kindergarten	29	24 (83%)	2 (7%)	3 (10%)	
Grade 1	27	23 (85%)	1 (4%)	3 (11%)	
Grade 2	26	22 (85%)	1 (4%)	3 (11%)	
Grade 3	30	28 (93%)	0 (0%)	2 (7%)	
Grade 4	28	25 (89%)	0 (0%)	3 (11%)	
Grade 5	41	35 (85%)	0 (0%)	6 (15%)	
Grade 6	55	45 (82%)	0 (0%)	10 (18%)	
Grade 7	57	50 (88%)	0 (0%)	7 (12%)	
Grade 8	58	50 (86%)	0 (0%)	8 (14%)	
Grade 9	68	58 (85%)	1 (1%)	9 (13%)	
Grade 10	96	83 (86%)	1 (1%)	12 (13%)	
Grade 11	83	72 (86%)	1 (1%)	10 (12%)	
Grade 12	82	60 (73%)	4 (5%)	18 (22%)	
Gender					0.09
Male	427	371 (87%)	6 (1%)	50 (12%)	
Female	253	204 (81%)	5 (2%)	44 (17%)	
School location					0.34
Urban	427	356 (84%)	6 (1%)	65 (15%)	
Rural	253	219 (87%)	5 (2%)	29 (11%)	
Spherical equivalent of more astigmatic eye (Diopters)					0.78
≤ −6	174	145 (83%)	2 (1%)	27 (16%)	
> −6, ≤ −5	75	59 (79%)	1 (1%)	15 (20%)	
> −5, ≤ −4	90	74 (82%)	1 (1%)	15 (17%)	
> −4, ≤ −3	84	71 (85%)	2 (2%)	11 (13%)	
> −3, ≤ −2	54	46 (85%)	2 (4%)	7 (13%)	
> −2, ≤ −1	67	60 (90%)	1 (2%)	6 (9%)	
> − 1, ≤ −0.5	35	29 (83%)	1 (3%)	5 (14%)	
> − 0.5, < 0.5	48	45 (94%)	1 (2%)	2 (4%)	
≥ 0.5, < 1.0	20	16 (80%)	1 (5%)	3 (15%)	
≥ 1.0	33	30 (91%)	0 (0%)	3 (9%)	

Among the children with astigmatism, we evaluated the factors associated with severity of astigmatism based on cylinder power. In multivariate analysis that included grade, gender, school location and spherical equivalent, male gender was significantly associated with more severe astigmatism. The mean cylinder power was 2.21 D in male students as compared to 2.06 D in female students (*p* = 0.04). Myopic or hyperopic refractive error was also significantly associated with more severe astigmatism, the mean cylinder power being 2.50 D for students with myopic − 6.0 D or worse, and 2.26 D for students with hyperopia 1.0 D or greater as compared to 1.83 for those with spherical equivalent of 0.5 to 1.0 D (*p* = 0.007, Table 7).

**Table 7:** Multivariate analyses for the factors associated with degree of astigmatism among those with astigmatism (*N* = 680)

		Multivariate Model 1: without including spherical equivalent *		**Multivariate Model 2: including spherical equivalent**^**§**^	
Demographics	N	Mean cylinder in diopter (SE)	*P*-value	Mean cylinder in diopter (SE)	*P*-value
Grade			0.91		0.93
Kindergarten	29	2.14 (0.18)		2.13 (0.19)	
Grade 1	27	2.31 (0.19)		2.38 (0.19)	
Grade 2	26	2.12 (0.19)		2.18 (0.20)	
Grade 3	30	2.14 (0.18)		2.15 (0.18)	
Grade 4	28	2.24 (0.18)		2.27 (0.18)	
Grade 5	41	2.03 (0.15)		2.02 (0.18)	
Grade 6	55	2.34 (0.13)		2.26 (0.15)	
Grade 7	57	2.03 (0.13)		1.96 (0.13)	
Grade 8	58	2.15 (0.13)		2.07 (0.13)	
Grade 9	68	2.23 (0.12)		2.13 (0.13)	
Grade 10	96	2.21 (0.10)		2.10 (0.11)	
Grade 11	83	2.29 (0.11)		2.10 (0.12)	
Grade 12	82	2.24 (0.11)		2.06 (0.12)	
Gender			0.08		0.04
Male	427	2.26 (0.06)		2.21 (0.05)	
Female	253	2.12 (0.05)		2.06 (0.06)	
School location			0.57		0.57
Urban	427	2.17 (0.06)		2.12 (0.06)	
Rural	253	2.21 (0.06)		2.16 (0.07)	
Spherical equivalent of more astigmatic eye (diopters)					0.007
≤ −6	174			2.50 (0.09)	
> −6, ≤ −5	75			2.24 (0.12)	
> −5, ≤ −4	90			2.09 (0.11)	
> −4, ≤ −3	84			2.14 (0.11)	
> −3, ≤ −2	54			2.19 (0.14)	
> − 2, ≤ −1	67			2.25 (0.12)	
> −1, ≤ −0.5	35			1.92 (0.17)	
> − 0.5, < 0.5	48			1.98 (0.14)	
≥ 0.5, < 1.0	20			1.83 (0.22)	
≥ 1.0	33			2.26 (0.17)	

*Multivariate model 1 included grade, gender and school location

^§^Multivariate model 2 included grade, gender, school location and spherical equivalent

## Discussion

This large school-based study evaluated the prevalence, severity, and type of astigmatism in school-aged students in the YiWu city of eastern China. The study found that the overall prevalence rate of astigmatism was 14% for astigmatism of 1.5 D or greater and 2% for high astigmatism of 3.0 D or greater. The majority of astigmatism (~ 85%) was with-the-rule. The prevalence of astigmatism increased with grade, and was higher in the male students. The myopic or hyperopic refractive error was independently associated with higher prevalence rate and severity of astigmatism.

It is well-known that prevalence of astigmatism varies with race and ethnicity [4, 5, 7, 8]. Previous studies using different cutpoint of cylinder power reported various high prevalence rates of astigmatism in Chinese preschool children and school-age students [7–12]. The study in Hongkong preschool children (mean age of 56 months) reported 55.8% of astigmatism 0.5 D or greater, 21.1% of astigmatism 1.0 D or greater, and 2.2% of astigmatism 2.0 D or greater [10]. Studies in Singapore reported prevalence rate of 19.2% for astigmatism 1.0 D or greater in school children [12], and 23.2% of astigmatism 1.0 D or greater in teenage high school students [13]. A study in Taiwan reported 32.6% prevalence rate of astigmatism 1.0 D or greater in school children [11]. In studies of astigmatism in mainland China, prevalence rate of astigmatism varied substantially. A study in Central China of Anyang city reported 17.4% prevalence rate of astigmatism of 1.0 D or greater, and 5.9% prevalence rate of 1.5 D or great in urban 12-year-old school students [7]. A study in urban South China of Guangzhou reported 42.7% of astigmatism of 0.75 D or greater in 5–15 year-old students [14], another study in rural southern China of Yangxi county reported 25.3% of astigmatism of 0.75 D or greater in 13–15 year-old students [15]. The study in urban of northern China reported 7.5% of astigmatism of 0.75 D or greater in 5–15 year-old students [16]. Our study had a higher prevalence rate of astigmatism than previous studies in Chinese population with prevalence rate of 75.9% of astigmatism 0.5 D or greater, 33% of astigmatism of 1.0 D or greater, and 14.2% of astigmatism of 1.5 D or greater, and 2.2% of astigmatism of 3.0 D or greater. These differences in astigmatism prevalence rate may be due to the differences in the characteristics of participating students (age, refractive error, etc.) and the method of measuring cylinder power. We believe this high prevalence rate of astigmatism in our study may be due to the high prevalence rate of myopia (71% with spherical equivalent of − 0.5 D or worse) in our study population.

Our study found that male students had significantly higher prevalence rate of astigmatism (16.1% vs. 11.8%) and high astigmatism (2.8% vs. 1.4%) than female students, but there was no significant difference in types of astigmatism. In the multivariate analyses that were adjusted by the spherical equivalent levels, the prevalence rate in male students were 2.2 times that of female students for both astigmatism and high astigmatism. The Multi-Ethnic Pediatric Eye Disease Study (MEPEDS) found prevalence of astigmatism (≥ 1.5 D) was higher in Hispanic male (18.5%) than female (14.9%) (*p* = 0.02 adjusting for age) [17]. A study in Singapore high school students also found that astigmatism (≥ 0.5 D) was higher in male students than female students (61.2% vs. 55.5%, *p* = 0.08) [13]. However, no significant gender difference in astigmatism prevalence was found in Hongkong preschool children [9], in 12-year-old Chinese students of Anyang Childhood Eye Study [7], in non-Hispanic White and Asian children of MEPEDS study [5], and in the Vision In Preschoolers (VIP) study [4]. On the contrary, two previous studies in China found higher prevalence rate of astigmatism in female students than male students [14, 16]. In a previous publication from this study, we reported myopia rate was 1.5 times higher in female students than male students [6], it is intriguing that prevalence rate of astigmatism these male students was two times that of female students. This gender difference in astigmatism prevalence warrants future investigation through the longitudinal studies.

Our study found that the majority (85%) of astigmatism was with-the-rule (defined as cylinder axis 90 ± 15 degrees). The dominance of with-the-rule as a type of astigmatism is consistent with previous studies in Chinese children in Xiamen of southeastern China (72% with-the-rule) [18], in Guangxi of southern China (83%) [8], Anyang of Northern China (58%) [7], Taiwan (83% in 1995 and 90% in 2000) [11], Singapore (73%) [13], and Hongkong (57%) [19]. However, a study in Australia found against-the-rule was the dominant type of astigmatism in Australia school children [20]. The factors associated with type of astigmatism was not well studied. However, our study did not find any significant factors associated with the type of astigmatism.

Our study clearly demonstrated the non-linear association between spherical equivalent and astigmatism (Fig. 2). Children with myopic or hyperopic refractive error had higher prevalence of astigmatism than children with emmetropic refractive error. This non-linear associations was similar to findings from the previous large studies of MEPEDS [17], Anyang Childhood Eye Study [7] and the VIP study [4]. Because these studies are all cross-sectional, we can not determine their causal relationship between spherical equivalent and astigmatism. Future longitudinal studies are needed to investigate their possible causal relationship.

In this study, we found the older age and higher grade were associated with higher prevalence rate of astigmatism. However, the association was not significant after adjusting the spherical equivalent, suggesting that significant refractive error contributed to the increasing prevalence rate of astigmatism with higher grade. The higher rate of astigmatism in our study might be due to the high prevalence rate of myopia in this study.

Strengths of the study include the large sample size and the wide age range of students from kindergarten to high school students. A possible limitation includes the use of cylinder measures from NIDEK non-cycloplegic autorefractor (Model: AR-1 s, Japan) to determine astigmatism and to assess the spherical equivalent as a risk factor for astigmatism. The non-cycloplegic autorefraction could potentially bias our estimate of cylinder power and prevalence rate of astigmatism. However, a study did not find any statistically significant difference in axis and cylinder power of astigmatism measured before and after cycloplegia in the 4 to 17 year old children [21]. Another study reported higher rate of astigmatism when measured using autorefraction (ARK-30) than with retinoscopy (42.7% vs. 33.6%) in children 5 to 15 years old and this difference was mainly due to the mild or moderate forms of astigmatism [14]. Since we used the higher cut points (≥ 1.5 D and ≥ 3.0 D) for defining astigmatism, using cylinder power from autorefraction unlikely substantially bias the estimate of astigmatism prevalence. Another limitation of this study is the lack of further investigations on the children with high degrees of astigmatism. Further evaluation using corneal topography or pentacam might be helpful to determine whether keratoconus as one the possible causes of astigmatism.

## Conclusions

Our study found that astigmatism is common in Chinese school-age children and prevalence rate of astigmatism increases with grade, likely due to significant refractive error. The majority of astigmatism was with-the-rule. Male gender and myopic or hyperopic refractive error were significantly associated with higher prevalence and severity of astigmatism. Future longitudinal studies are needed to investigate the possible causal relationship between myopic or hyperopic refractive error and astigmatism, and to evaluate whether intervention on refractive error can reduce the development of astigmatism.

## Data Availability

De-identified data are available upon request to the first author.

## Abbreviations

SE: Spherical Equivalent
D: Diopters
OR: Odds Ratio
CI: Confidence Interval
MEPEDS: Multi-Ethnic Pediatric Eye Disease Study
VIP: Vision In Preschoolers

## References

[1] Abrahamsson M, Sjostrand J (2003). Astigmatic axis and amblyopia in childhood. Acta Ophthalmol Scand.

[2] Fulton AB, Hansen RM, Petersen RA (1982). The relation of myopia and astigmatism in developing eyes. Ophthalmology.

[3] Gwiazda J, Grice K, Held R, McLellan J, Thorn F (2000). Astigmatism and the development of myopia in children. Vis Res.

[4] Huang JY, Maguire MG, Ciner E (2014). Risk factors for astigmatism in the vision in preschoolers study. Optometry Vision Sci.

[5] Wen G, Tarczy-Hornoch K, McKean-Cowdin R (2013). Prevalence of myopia, hyperopia, and astigmatism in non-Hispanic white and Asian children: multi-ethnic pediatric eye disease study. Ophthalmology.

[6] Wang J, Ying GS, Fu X (2020). Prevalence of myopia and vision impairment in school students in eastern China. BMC Ophthalmol.

[7] Li H, Li SM, Liu LR (2019). Astigmatism and its components in 12-year-old Chinese children: the Anyang childhood eye study. Br J Ophthalmol.

[8] Xiao X, Liu WM, Ye YJ (2014). Prevalence of high astigmatism in children aged 3 to 6 years in Guangxi, China. Optometry Vision Sci.

[9] Fan DS, Rao SK, Cheung EY, Islam M, Chew S, Lam DS (2004). Astigmatism in Chinese preschool children: prevalence, change, and effect on refractive development. Br J Ophthalmol.

[10] Leung TW, Lam AK, Deng L, Kee CS (2012). Characteristics of astigmatism as a function of age in a Hong Kong clinical population. Optom Vis Sci.

[11] Shih YF, Hsiao CK, Tung YL, Lin LL, Chen CJ, Hung PT (2004). The prevalence of astigmatism in Taiwan schoolchildren. Optom Vis Sci.

[12] Tong L, Saw SM, Carkeet A, Chan WY, Wu HM, Tan D (2002). Prevalence rates and epidemiological risk factors for astigmatism in Singapore school children. Optom Vis Sci.

[13] Quek TP, Chua CG, Chong CS (2004). Prevalence of refractive errors in teenage high school students in Singapore. Ophthalmic Physiol Opt.

[14] He M, Zeng J, Liu Y, Xu J, Pokharel GP, Ellwein LB (2004). Refractive error and visual impairment in urban children in southern China. Invest Ophthalmol Vis Sci.

[15] He M, Huang W, Zheng Y, Huang L, Ellwein LB (2007). Refractive error and visual impairment in school children in rural southern China. Ophthalmology.

[16] Zhao J, Pan X, Sui R, Munoz SR, Sperduto RD, Ellwein LB (2000). Refractive error study in children: results from Shunyi District, China. Am J Ophthalmol.

[17] Fozailoff A, Tarczy-Hornoch K, Cotter S (2011). Prevalence of astigmatism in 6- to 72-month-old African American and Hispanic children: the multi-ethnic pediatric eye disease study. Ophthalmology.

[18] Zhan MZ, Saw SM, Hong RZ (2000). Refractive errors in Singapore and Xiamen, China--a comparative study in school children aged 6 to 7 years. Optom Vis Sci.

[19] Park SW, Sok JY, Park YG (2008). The effect of surgical correction of epiblepharon on astigmatism in children. J Pediatr Ophthalmol Strabismus.

[20] Huynh SC, Kifley A, Rose KA, Morgan IG, Mitchell P (2007). Astigmatism in 12-year-old Australian children: comparisons with a 6-year-old population. Invest Ophthalmol Vis Sci.

[21] Goyal S, Phillips PH, Rettiganti M, Gossett JM, Lowery RS (2018). Comparison of the effect of Cycloplegia on astigmatism measurements in a pediatric amblyopic population: a prospective study. J Pediatr Ophthalmol Strabismus.

